# Redox-mediated quorum sensing in plants

**DOI:** 10.1371/journal.pone.0182655

**Published:** 2017-09-13

**Authors:** Alexandra W. Fuller, Phoebe Young, B. Daniel Pierce, Jamie Kitson-Finuff, Purvi Jain, Karl Schneider, Stephen Lazar, Olga Taran, Andrew G. Palmer, David G. Lynn

**Affiliations:** 1 Departments of Biology and Chemistry, Emory University, Atlanta, GA, United States of America; 2 Gottwald Science Center, University of Richmond, Richmond, VA, United States of America; 3 Department of Biological Sciences, Florida Institute of Technology, Melbourne, FL, United States of America; National Taiwan University, TAIWAN

## Abstract

The rhizosphere, the narrow zone of soil around plant roots, is a complex network of interactions between plants, bacteria, and a variety of other organisms. The absolute dependence on host-derived signals, or xenognosins, to regulate critical developmental checkpoints for host commitment in the obligate parasitic plants provides a window into the rhizosphere’s chemical dynamics. These sessile intruders use H_2_O_2_ in a process known as semagenesis to chemically modify the mature root surfaces of proximal host plants and generate *p*-benzoquinones (BQs). The resulting redox-active signaling network regulates the spatial and temporal commitments necessary for host attachment. Recent evidence from non-parasites, including *Arabidopsis thaliana*, establishes that reactive oxygen species (ROS) production regulates similar redox circuits related to root recognition, broadening xenognosins’ role beyond the parasites. Here we compare responses to the xenognosin dimethoxybenzoquinone (DMBQ) between the parasitic plant *Striga asiatica* and the non-parasitic *A*. *thaliana*. Exposure to DMBQ simulates the proximity of a mature root surface, stimulating an increase in cytoplasmic Ca^2+^ concentration in both plants, but leads to remarkably different phenotypic responses in the parasite and non-parasite. In *S*. *asiatica*, DMBQ induces development of the host attachment organ, the haustorium, and decreases ROS production at the root tip, while in *A*. *thaliana*, ROS production increases and further growth of the root tip is arrested. Obstruction of Ca^2+^ channels and the addition of antioxidants both lead to a decrease in the DMBQ response in both parasitic and non-parasitic plants. These results are consistent with Ca^2+^ regulating the activity of NADPH oxidases, which in turn sustain the autocatalytic production of ROS via an external quinone/hydroquinone redox cycle. Mechanistically, this chemistry is similar to black and white photography with the emerging dynamic reaction-diffusion network laying the foundation for the precise temporal and spatial control underlying rhizosphere architecture.

## Introduction

The plant rhizosphere is so richly populated by mutualistic associations that it has been dubbed the second plant genome [1]. With an estimated 10^11^ microbial cells/gm of root tissue [2] containing as many as 10^4^ different species [3], the plant root must recruit, maintain, and defend this complex extracellular microbiome. Bacterial colonization depends on a rich and diverse chemical language where the rates of exudation, the inherent physical and biological stability of the agents, and the differential responses of members of the rhizosphere all contribute to this information rich and biologically dynamic signaling landscape [4–8]

Beyond their role in regulating plant-microbial associations, insights into the plant’s contribution to this signaling landscape has emerged from studies on: (i) host recognition by parasitic plants [9–12], (ii) kin recognition/selection [13–16], and (iii) allelopathy [17,18]. Our understanding of the redox dynamics of these chemical networks has expanded significantly with the discovery of *semagenesis* [19,20]. In this process, reactive oxygen species (ROS) exuded from the root tip of the parasite initiates a mild ‘wound’ response that ultimately leads to oxidation of a prospective host’s cell wall phenols into *p*-benzoquinones (BQs) [19,21]. Persistent exposure of the parasite root meristem to BQs is necessary and sufficient to induce organogenesis of the parasite’s attachment organ, the haustorium [9,22,23], and serves as a signature for viable host roots.

**Fig 1:**
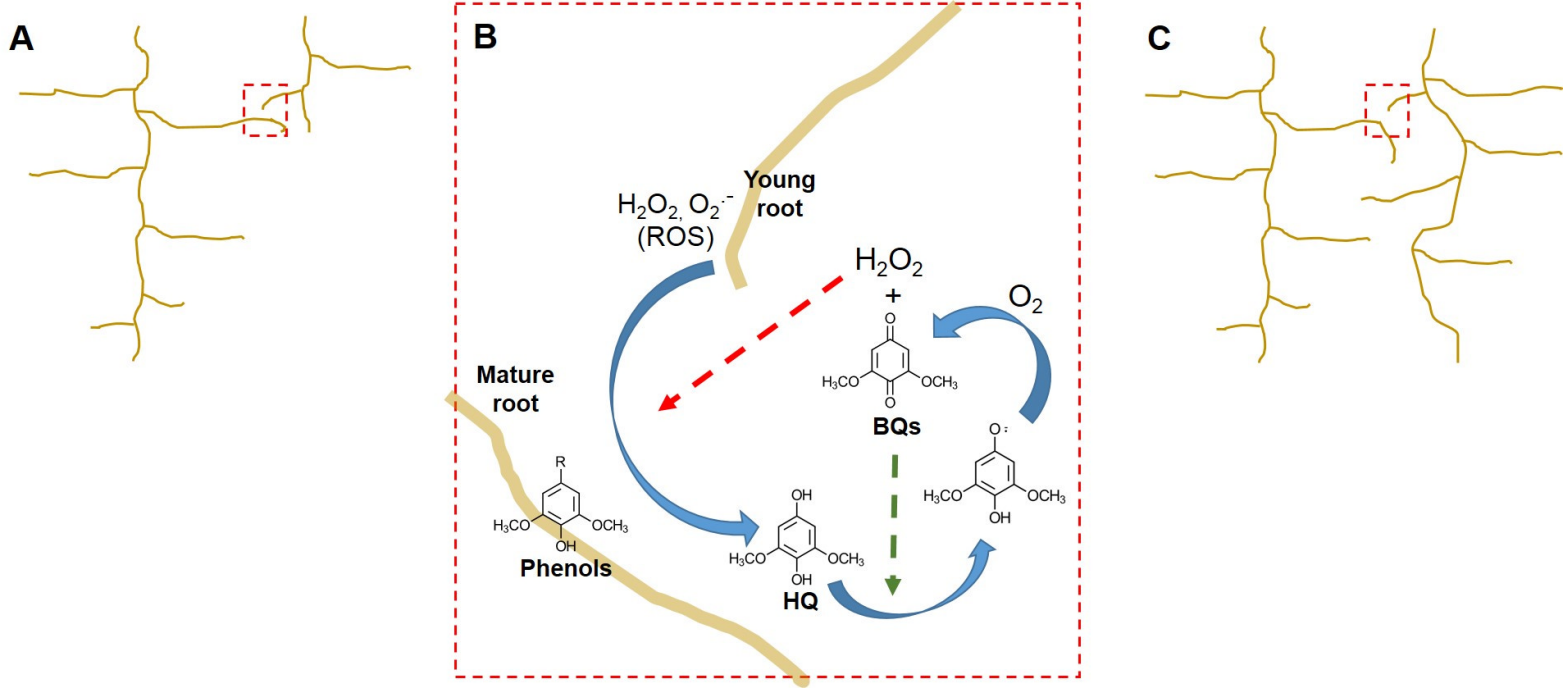
ROS/phenol/BQ reaction network in plants. **Simple model for ‘quorum’ or collision sensing in Arabidopsis.** (A) A growing lateral root from a young seedling encounters an established plant (red box). (B) Within a certain distance, ROS produced by the tip of the growing seedling contributes to oxidation of cell wall associated phenols to yield BQs (red arrow). DMBQ is shown here as an example. These BQs accumulate, contribute to the autocatalytic production of new ROS intermediates, amplify the signal (green arrow). (C) Evidence of an established root system arrests root elongation in a quorum-like process, regulating global root system architecture.

Evidence that non-parasitic members of the Eudicotidae clade, including *Nicotiana tabacum* and *Arabidopsis thaliana* [24], also produce ROS at the growing root tip suggested that semagenesis may serve a broader role in the rhizosphere. Indeed, the same chemical network which generates BQs via the oxidation of host derived phenols by parasite derived ROS, could reasonably be recreated by replacing the parasite root with any root tip. We propose that the pairing of the roots between non-parasites would provide a mechanism for younger roots to detect a more established neighbor and adjust root system architectures (RSA) accordingly (Fig 1).

The half-life of hydrogen peroxide (H_2_O_2_) in biotic matrices is estimated to be on the order of milliseconds, with other ROS intermediates decomposing even faster [25–27], consistent with the oxidation of phenols occurring only within 50 μm of the growing root tip [9,22,23]. In the mixed-microbial biofilms where H_2_O_2_ is used as an antimicrobial agent by streptococci, its diffusion zone may be limited to 4 μm [28], providing an even more precise ruler in those matrices. In addition, the hydroquinone (H_2_Q) intermediates, which accumulate during the reaction between root-tip derived ROS and cell wall phenols, can further react with molecular oxygen to generate additional H_2_O_2_ (Fig 1B). Thus H_2_O_2_ concentrations may amplified chemically *outside* the root cells [29] as shown in Fig 1. Here, H_2_O_2_ initiates phenol decomposition yielding the hydroquinone, H_2_Q, which in the presence of oxygen generates BQ and more H_2_O_2_ [30]. In this scenario, the concentration and spatial distribution of H_2_Q, BQ, and ROS, all critical members of this autocatalytic cycle, are co-dependent and defined by the oxygen tension in the rhizosphere.

We now report a simple assay to evaluate the impact of root density on growth of *A*. *thaliana* seedlings and provide evidence that BQ exposure recapitulates the root density phenotype [15,31,32]. Further, we report a comparative study of calcium fluctuations that connect BQ exposures in Striga and Arabidopsis spp. Taken together, these results support semagenesis as an active quorum sensing-like strategy. Further, since both pathogenic and mutualistic *Rhizobium spp* depend on the very phenols involved in this dynamic chemical network, the redox circuit should also impact their symbiotic behaviors. We argue that these dynamic redox chemical networks provide critical environmental information to a broad array of inhabitants of the rhizosphere. Finally, we propose that semagenesis provides quorum sensing (QS) information on root density, a process that has been effectively re-deployed by parasitic members for the purposes of host detection.

## Materials and methods

### Materials

Agarose, sucrose, and MES were obtained from Merck (Darmstadt, Germany). H_2_DCF-DA was purchased from Molecular Probes (Carlsbad, CA). All other chemicals were purchased from Sigma Aldrich (St. Louis, MO) or Caisson Labs (Logan, UT).

### Pre-treatment & germination of Striga

*Striga asiatica* seeds were obtained from Drs. R.E. Eplee and Rebecca Norris (U.S. Department of Agriculture, Witchweed Methods Development Laboratory; Oxford, NC). All *S*. *asiatica* work was done under the auspices of the USDA quarantine licenses awarded to Emory University. *S*. *asiatica* seedlings were pre-treated by washing the seedlings in the following order: 3% chromic acid for 3 minutes, a solution of 1% Tween-20 and 7% bleach for 7 minutes, and finally 70% ethanol for 1 minute. The seeds were then rinsed and placed in ddH_2_O for 10–14 d in capped Erlenmeyer flasks. Following incubation, the seeds were germinated by 24 h exposures to 10^−9^ M strigol in 0.1 mM KCl [21].

### Sterilization and growth of Arabidopsis seedlings

*Arabidopsis thaliana* seedlings (50 μl) were sterilized by successive 3 minute treatments with: (i) 5% Bleach + 0.05% Tween-20 solution and (ii) 70% ethanol. Samples were rinsed 3 times with an equal volume of sterile distilled water after both treatments. Sterile seeds were suspended in 1 mL of dH_2_O and spread on MS plates (0.25X Murashige & Skoog salts, 1% sucrose, and 0.8% agar, pH: 5.6). Seedlings were plated with approximately 50 seedlings/plate, stored in the dark at 4°C for 3–4 days, and then grown for 5–7 days in a temperature controlled growth chamber (22°C) with a 16 h photoperiod.

### Haustorial induction and inhibition

All Striga experiments are initiated one day post-germination. Striga seedlings were placed in 6 well plates (30 seedlings/well) with 5 mL of 0.1 mM KCl, 10 μM DMBQ, and the indicated concentration of LaCl_3_. Seedlings are scored as having successfully formed a haustorium if the root tip displays both swelling as well as haustorial hair formation. The entire process of haustorium development requires 24 hours, so hair formation can only be observed in two day-old seedlings. For timed exposure assays, the treatment was applied for the indicated time interval, after which the liquid was removed from the well and the seedlings were washed three times with 0.1 mM KCl. Seedlings were then placed in 5 mL of 0.1 mM KCl and scored for haustorial development at 24 hours. Reversibility of LaCl_3_ inhibition was accomplished by triplicate washings with 100 μM CaCl_2_ followed by returning seedlings to the 0.1 mM KCl buffer. Unless otherwise stated, the data are expressed as the average of three experiments ± standard deviation.

### H_2_DCF-DA imaging for ROS

Seedlings of *S*. *asiatica* and *A*. *thaliana* were analyzed for ROS production using H_2_DCFDA as a fluorescent probe as previously described [19]. Bovine liver catalase was used to scavenge ROS in the media by adding 250 units/ml of culture, incubating for 1 hour at ambient temperature, and inactivation by heating the sample to 100°C on a heat block for 15 minutes. Seedling density varied from 1 seedling per well to a maximum of 15 seedlings per well (5 seedlings/1 ml).

### Ca^2+^ imaging in *Striga asiatica*

One day-old seedlings of *Striga asiatica* were loaded with 50 μM Fluo-4 AM for 20 minutes at 4°C then returned to room temperature for 10 minutes. Seedlings were then washed in triplicate with ddH2O, transferred to a well of an 8 well microscope slide, resuspended in 250 μl of phosphate buffer (pH: 6.1) and imaged for basal Ca^2+^ via an epifluorescent microscope at set time points (Ex: 488/Em: 535 nm). The effect of DMBQ was evaluated by the addition of sufficient compound from stock to produce a 10 μM solution. Images were collected at the indicated time intervals to evaluate changes in fluorescence.

### LaCl_3_ exposures in *A*. *thaliana*

Following the growth period, *A*. *thaliana* seedlings were transferred to 6 or 12-well plates and treated with 3 ml of 0.25X MS media (0.25X Murashige & Skoog salts, 1% sucrose, and 0.8% agar, pH: 5.6) with or without LaCl_3_ (standard concentration of LaCl_3_ used was 10 μM). Seedling density varied from 1 seedling per well to a maximum of 15 seedlings per well.

### Imaging YC3.6 seedlings

7–10 day-old seedlings of *A*. *thaliana* expressing the fluorescence resonance energy transfer (FRET)-based Ca^2+^ sensor YC3.6 [33,34] were transferred to deep-well depression slides for visualization along with 1 ml of 0.25X Murashige & Skoog media. Ratiometric imaging was performed on a Nikon C1 laser scanning confocal microscope by exciting the cyan fluorescent protein (CFP) at 457 nm. Emission from CFP (λ_em_ = 473–505 nm) and from FRET-dependent Venus (λ_em_ = 526–536 nm) was collected at 480 nm and 528 nm.

### Data processing

Data analysis was conducted using the statistical program R (version 2.13.1). YC 3.6, Fluo4-AM, and H_2_DCF-DA fluorescence intensities were analyzed using Fiji (ImageJ).

## Results

### Calcium mediates haustorial organogenesis and ROS production in *S*. *asiatica*

BQ-initiated reduction of ROS production in *S*. *asiatica* has been correlated with down-regulation of the NADPH oxidase 1, *SaNOX1* [35], which contains two Ca^2+^-binding EF-hand domains. The activity of EF-hand containing NADPH oxidases has been positively correlated with Ca^2+^ concentrations in *A*. *thaliana*, supporting a link between BQ-mediated ROS production and calcium regulation [36–38]. Furthermore, given the morphological signatures of haustorial growth, swelling of the root tip and development of the haustorial hairs [39,40], the general role of calcium dynamics on ROS production [36,41–43], as well as polar growth of root hairs [34,41], we reasoned that Ca^+2^ signaling may integrate BQ exposures to internal biochemical events in plants.

**Fig 2:**
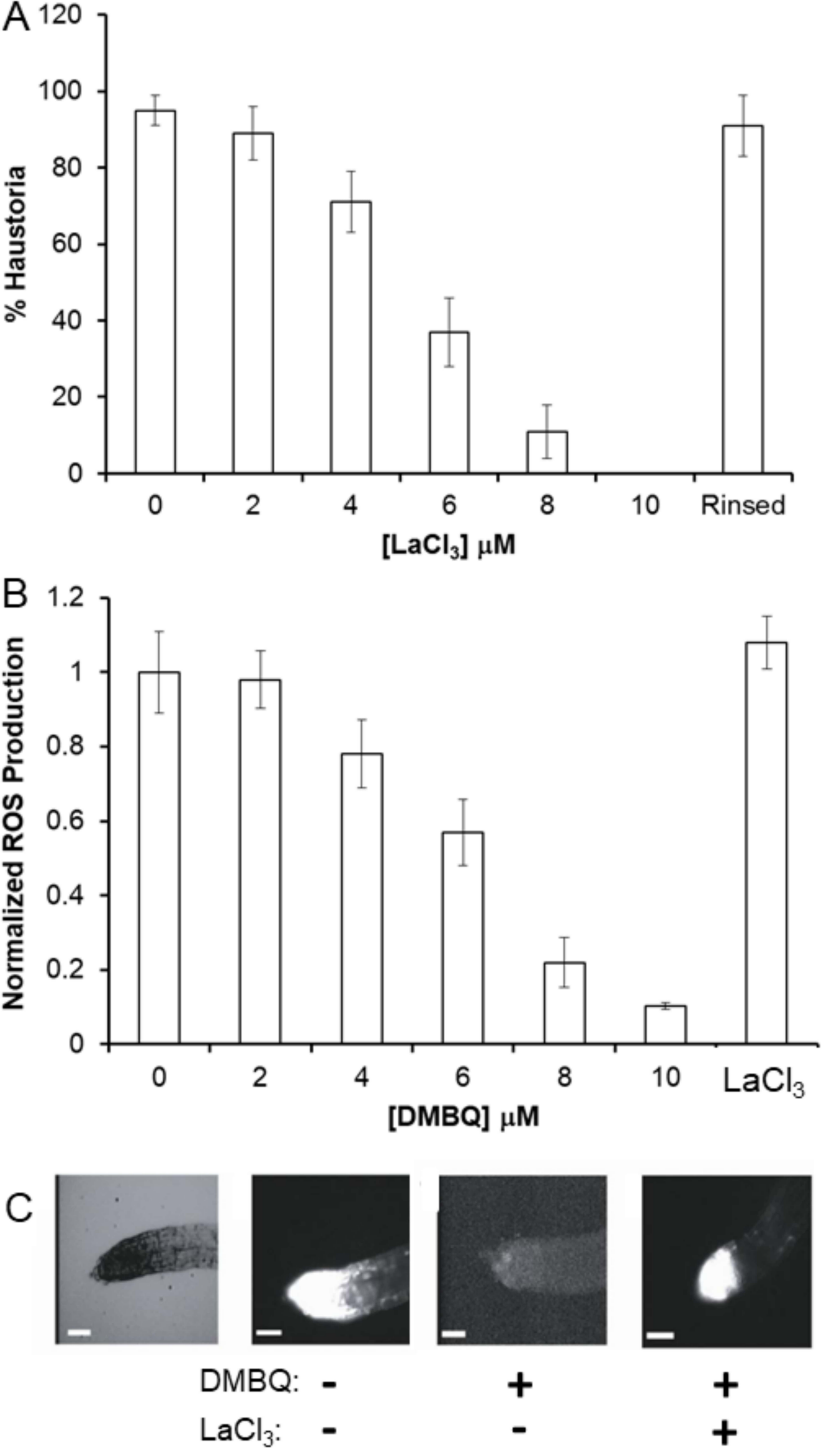
LaCl_3_ inhibits DMBQ perception. (A) Seedlings were treated with 10 μM DMBQ and the indicated concentration of LaCl_3_, then scored for haustorium formation after 24 h. ‘Rinsed’ seedlings were subsequently washed with 100 μM CaCl_2_, re-exposed to 10 μM DMBQ and scored for haustorium development after 24 h. (B) Seedlings treated with the indicated concentration of DMBQ for 2 hours were then incubated with H_2_DCF-DA and visualized for ROS production. LaCl_3_ seedlings were treated with 10 μM DMBQ and LaCl_3_ simultaneously, incubated for 2 hours then imaged for ROS production. (C) Bright field (light background) and H_2_DCF fluorescence images (dark background) of *S*. *asiatica* seedlings 2 hours after treatments with (+) or without (-) 10 μM DMBQ and/or LaCl_3_. Bar = 100 microns. All experiments conducted on day-old seedlings of *Striga asiatica* in triplicate with results expressed as average ± SD.

We began by evaluating whether the downstream response to BQ exposures is mediated through cellular calcium dynamics despite the differential phenotypic responses between parasites and non-parasites. Fig 2A shows the effect of the non-specific Ca^2+^-channel inhibitor lanthanum chloride (LaCl_3_) [44] on *S*. *asiatica* haustorial development when induced with 2,6-dimethoxy-*p*-benzoquinone (DMBQ). Under these conditions, half-maximal inhibition occurs at 5 μM LaCl_3_ but is reversed by rinsing the seedlings with CaCl_2_ (Fig 2A). The effects of DMBQ on ROS production can be observed using the cell-permeable ROS-sensitive fluorophore H_2_DCF-DA (2’,7’-dichlorodihydrofluorescein diacetate) [19]. DMBQ significantly reduces ROS accumulation in *S*. *asiatica* root tips (Fig 2B and 2C), and 10 μM LaCl_3_ effectively blocks DMBQ-induced down-regulation of ROS production. In the absence of these xenogenosins (BQs), no haustorial formation occurs.

**Fig 3:**
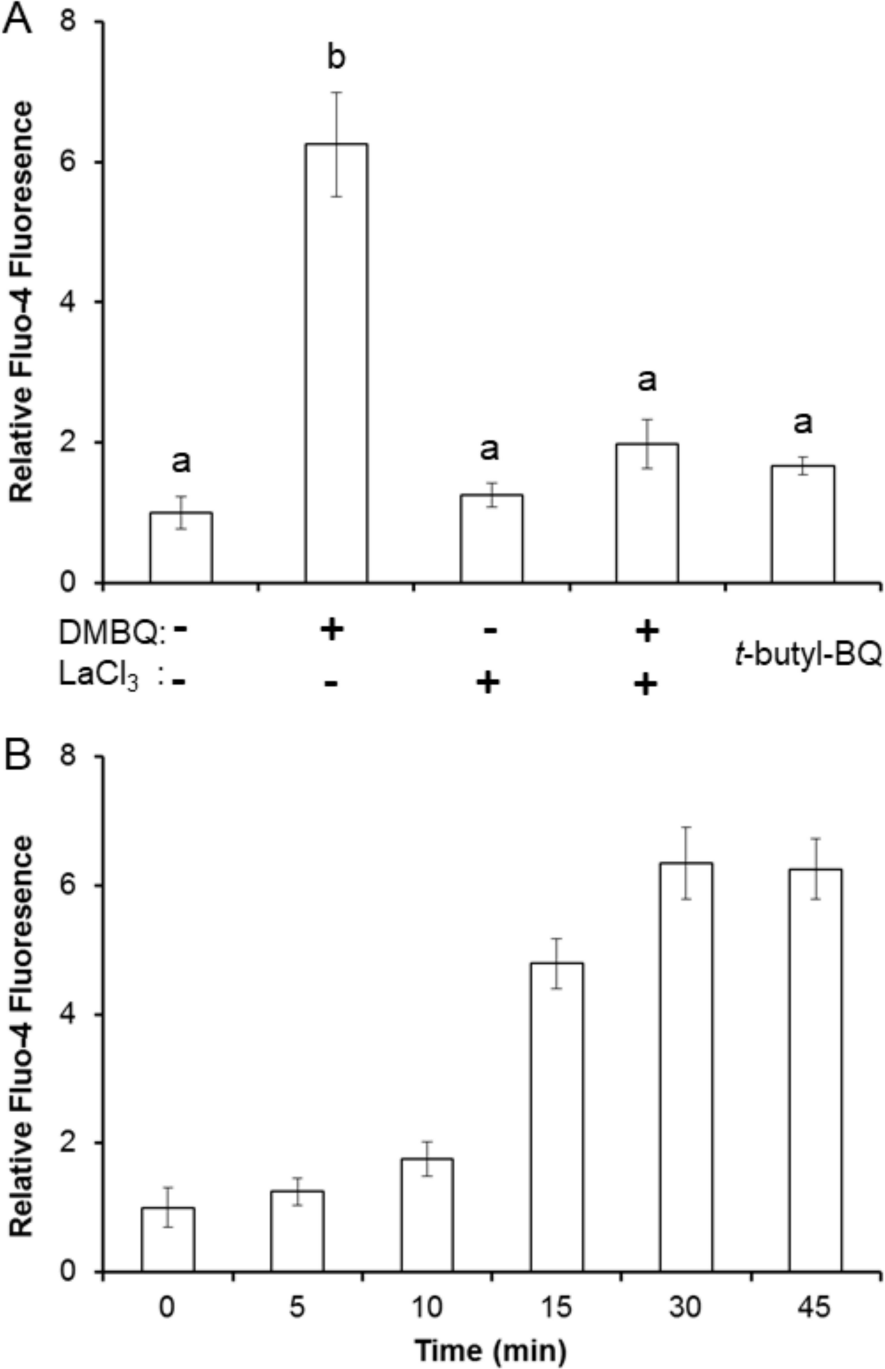
LaCl_3_ inhibits DMBQ perception in *Striga asiatica*. (A) Seedlings were treated with 10 μM DMBQ and/or 10 μM LaCl_3_ or with *t*-butyl-BQ for 1 hour then loaded with Fluo-4 AM and imaged for calcium-dependent fluorescence. (B) Seedlings were incubated with 10 μM DMBQ for the indicated time period then loaded with Fluo-4 AM and imaged for calcium dependent fluorescence. All experiments conducted on day-old seedlings of *S*. *asiatica* in triplicate with results expressed as average ± SD. Samples with different letters are statistically distinct from one another (*p*<0.05) based on a Tukey’s post-hoc test.

To directly visualize changes in cytoplasmic calcium concentrations ([Ca^2+^]_cyt_) in *S*. *asiatica* seedlings, we utilized the cell permeable fluorescent Ca^2+^-indicator Fluo4-AM [45]. Ten minute exposures to 10 μM DMBQ significantly increases Fluo4 fluorescence (Fig 3A) at the root tips of *S*. *asiatica* seedlings and this increase persists for at least 30 minutes (Fig 3B). LaCl_3_ inhibits terminal haustorial commitment and ROS production, consistent with a BQ-mediated influx of cytoplasmic calcium as a critical step early in the response pathways. The observation that the non-haustorial-inducing *tert*-butyl-*p*-benzoquinone does not increase [Ca^2+^]_cyt_ supports the effect being specific to haustorial inducing quinones.

### BQs regulate ROS and calcium flux in the root tips of *A*. *thaliana*

**Fig 4:**
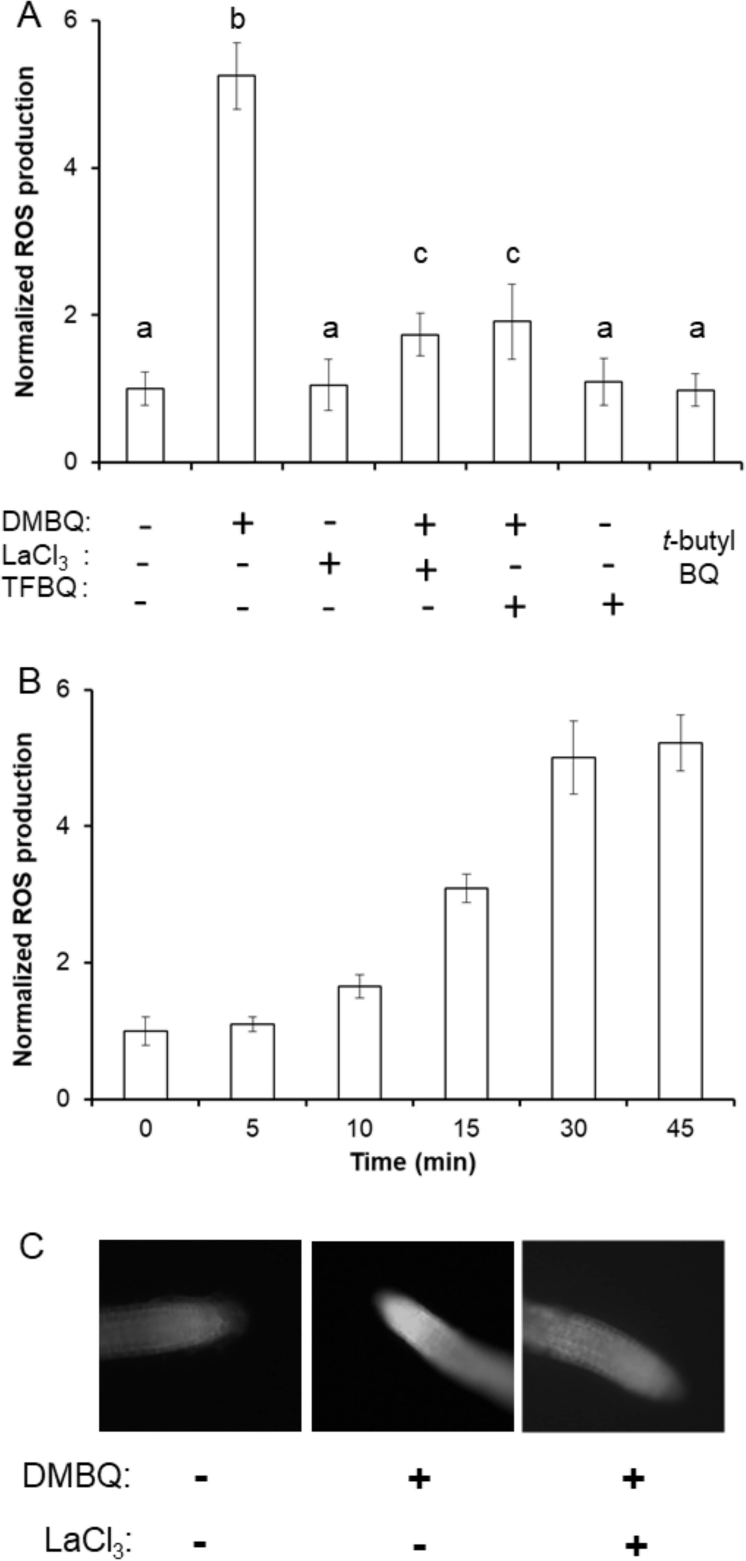
DMBQ induction of ROS depends on Ca^2+^ in *A*. *thaliana* seedlings. (A) Seedlings were treated with 10 μM DMBQ, LaCl_3_, and TFBQ as indicated for 1 hour then visualized for ROS production with H_2_DCF-DA. (B) Seedlings were treated with 10 μM DMBQ for the indicated period of time then visualized for ROS production with H_2_DCF-DA. Relative ROS production is normalized to DMBQ-free seedlings treated with H_2_DCF-DA, and all experiments are conducted in triplicate and expressed as average ± SD. Samples with different letters are statistically distinct from one another (*p*<0.05) based on a Tukey’s post-hoc test.

H_2_DCF-DA treatments confirm that DMBQ increases (Fig 4A), rather than decreases, ROS accumulation in the root tips of both *N*. *tabaccum* and *A*. *thaliana*. This is consistent with previous studies, that observed this increase in *N*. *tabaccum* seedlings with the ROS sensitive stain nitroblue tetrazolium [24]. Increased H_2_DCF-DA fluorescence is not seen with the non-haustorial inducing *t*-butyl BQ and is inhibited by LaCl_3_ (Fig 4A). Most importantly, ROS accumulation is inhibited by the BQ structural analog tetrafluorobenzoquinone (TFBQ), designed specifically to inhibit the redox events associated with haustorial-inducing BQs in *S*. *asiatica* (Fig 4A)[46,47]. DMBQ-induced production of ROS steadily increases over 1 hour (Fig 4B), consistent with the time previously shown to be required for haustorial induction in *S*. *asiatica* [19,47].

**Fig 5:**
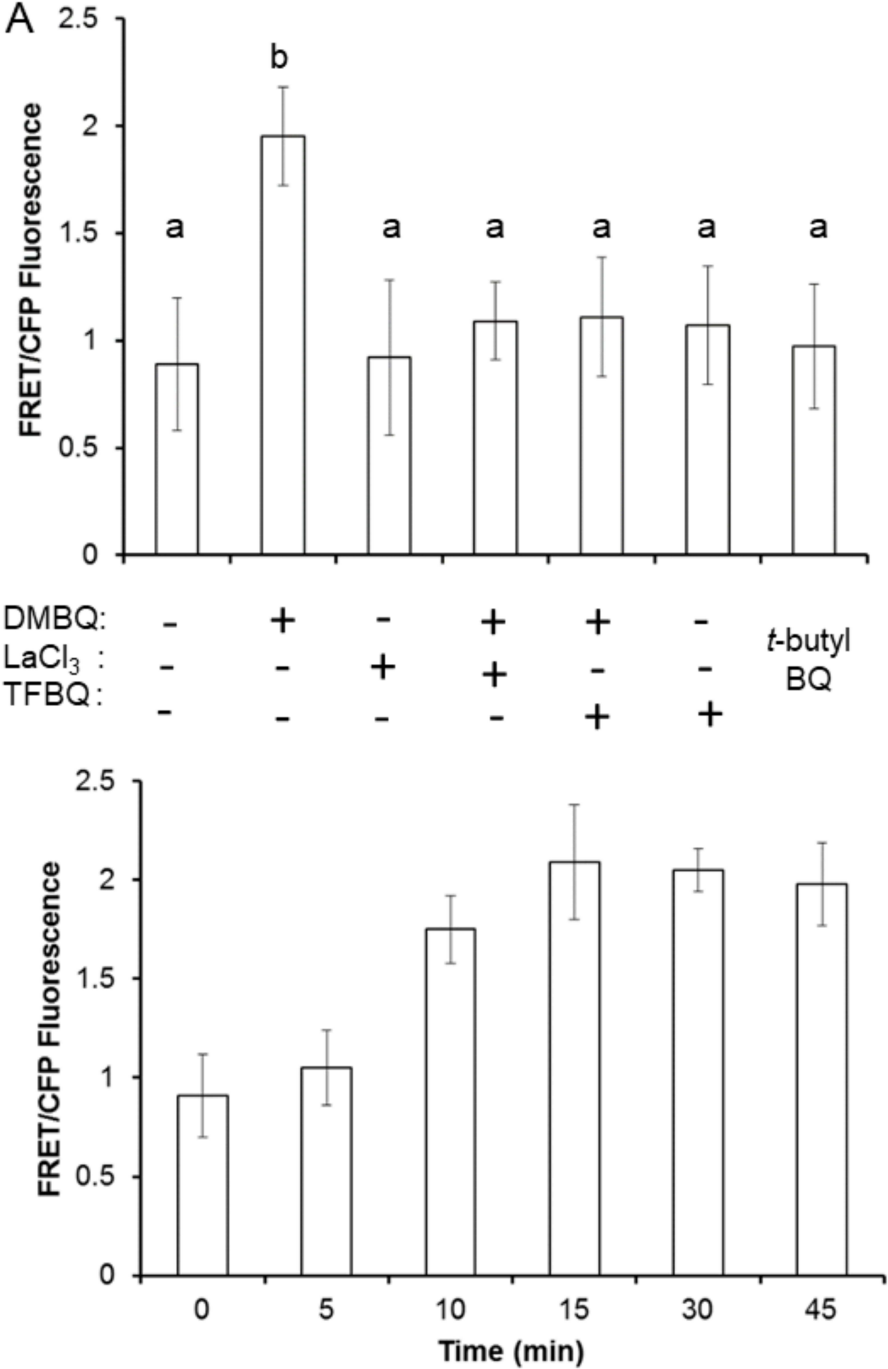
[Ca^2+^]_cyt_ -dynamics in A. thaliana YC3.6 seedlings in response to DMBQ. (A) Seedlings are treated with 10 μM DMBQ, LaCl_3_,.t-butyl BQ, and/or TFBQ as indicated for 1 hour then visualized for calcium changes. (B) Seedlings are loaded with 10 μM DMBQ and incubated for the indicated time before being scored for calcium-dependent fluorescence changes. All experiments are conducted on seedlings of *A*. *thaliana* YC3.6 in triplicate and the results are expressed as average ± SD. Samples with different letters are statistically distinct from one another (*p*<0.05) based on a Tukey’s post-hoc test.

Calcium dynamics in *A*. *thaliana* was specifically evaluated in strains expressing the YC3.6 FRET-based calcium reporter. This fusion protein consists of a cyan fluorescent protein (CFP - λ_em_ = 473–505 nm) linked by a calcium-sensitive calmodulin domain to a circularly permutated Venus (cpVENUS) fluorescent protein (λ_em_ = 526–536 nm)[34]. Calcium-binding to calmodulin brings these two fluorophores into contact, increasing resonance energy transfer from CFP to cpVENUS, creating a real-time, highly sensitive probe of [Ca^2+^]_cyt_. FRET-dependent VENUS fluorescence in this strain increases nearly two-fold on exposure to 10 μM DMBQ, consistent with an increase in [Ca^2+^]_cyt_. As in *S*. *asiatica*, increased [Ca^2+^]_cyt_ is inhibited by LaCl_3_ and TFBQ (Fig 5A). A statistically significant increase in [Ca^2+^]_cyt_ is detectable within 10 min of DMBQ exposure, plateaus after 15 min, and is sustained for at least 45 min (Fig 5B). Again, the non-haustorial-inducing *tert*-butyl-*p*-benzoquinone does not increase fluorescence (see Fig 5A).

### BQs simulate the effects of high root density

**Fig 6:**
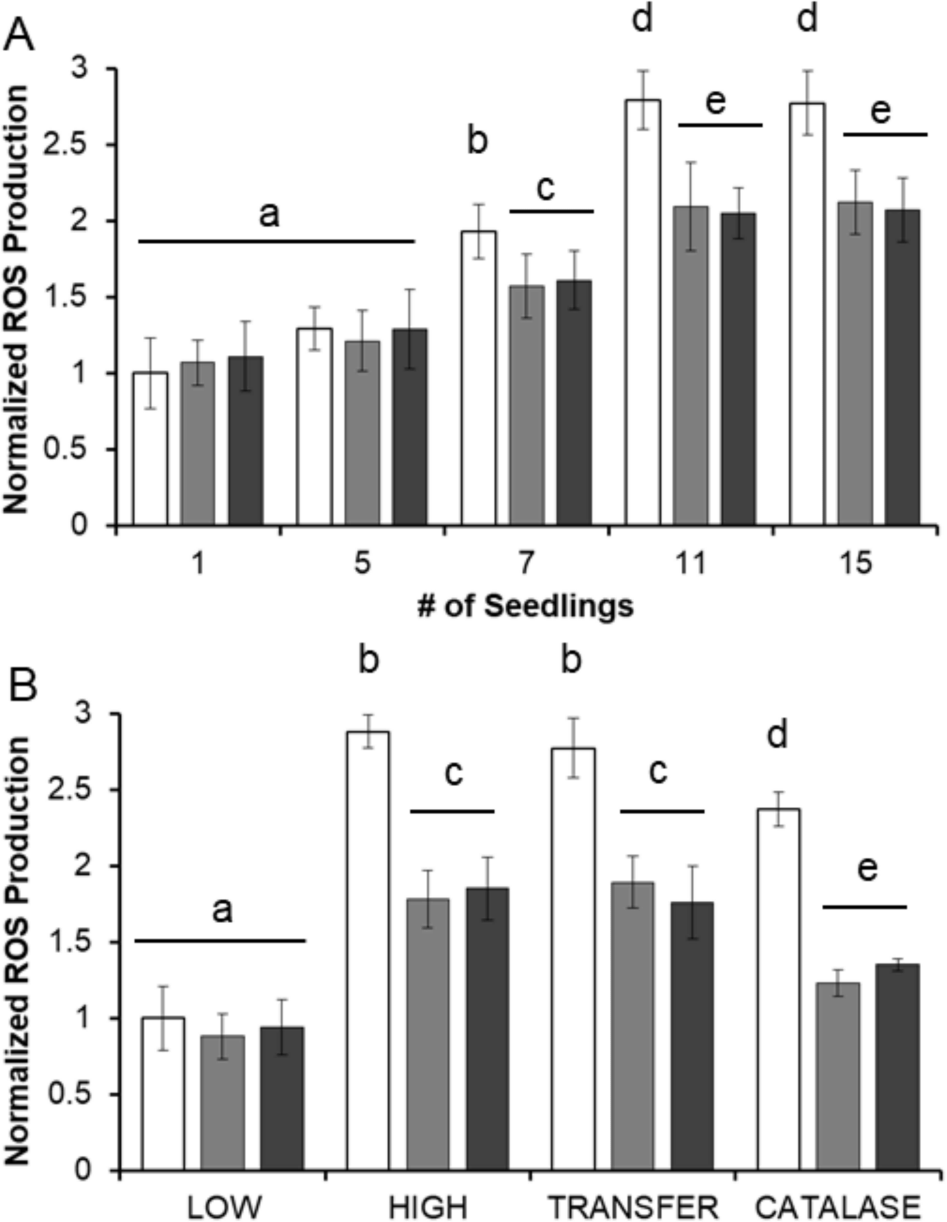
Root density impacts ROS production in *A*. *thaliana*. (A) The indicated number of seedlings are placed in 3 ml of MS media containing: 10 μM DMBQ (white), 10μM DMBQ and 10 μM LaCl_3_ (light grey), or 10 μM DMBQ and 10μM TFBQ (dark grey). After 2 hours the seedlings were visualized for ROS production with H_2_DCF-DA. (B) Individual seedlings were stored at ‘low density’ (1 seedling in 3 ml), ‘high density’ (1 seedling in 200 μl), or ‘transfer’ media (3 ml of media previously used to store 15 seedlings) with or without catalase treatment. Media was either supplemented with just DMSO stock (white) or with the addition of 10 μM LaCl_3_ (light grey) or 10μM TFBQ (dark grey). After 2 hours samples were visualized for ROS production with H_2_DCF-DA. All experiments conducted on 30 seedlings of *A*. *thaliana* in triplicate with results expressed as average ± SD. Samples with different letters are statistically distinct from one another (*p*<0.05) based on a Tukey’s post-hoc test.

To determine if semagenesis plays a functional role in non-parasite communities, a series of plant ‘density’ assays are outlined in Fig 6. *A*. *thaliana* seedlings are incubated at increasing densities (1, 5, 7, 11, or 15 seedlings) in 3 ml of MS media, and then evaluated for ROS production by H_2_DCF-DA. As seen in Fig 6A, ROS production positively correlates with seedling density. Density-dependent increases in ROS production are partially but not completely inhibited by LaCl_3_ and TFBQ in any of these treatments. Higher concentrations (>50 μM) of the inhibitors induce leaf chlorosis and/or necrosis within 24 h, limiting their usable concentration range. Placing a single seedling in 200 μl of buffer for 2 hours, the same density as 5 seedlings/ml, gave similar fluorescence intensities as with 15 seedlings in 3 ml (Fig 6B).

Oxidation of wall phenols is expected to yield a range of BQs with DMBQ as a representative example. While ROS are not stable in a biological matrix, the BQs, notably DMBQ, might accumulate in these matrices. To test that possibility, seedlings maintained at ‘low’ density (1 seedling in 3 ml) are incubated for 2 hours in ‘high’ density media recovered from separate solutions prepared by pre-incubating 5 seedlings/ml for 2 hours. ROS production increases in the transferred media and LaCl_3_ and TFBQ exposures again partially inhibit this increase (Fig 6B). However, it is possible that observed increases in ROS production in transferred media are due to carry over of ROS. To control for ROS transfer, the high-density media is treated for 1 h with catalase to scavenge H_2_O_2_ then inactivated by heat. As seen in Fig 6B, catalase-treated samples from ‘high density seedling’ media increase ROS production and are attenuated by both LaCl_3_ and TFBQ treatments, reflecting the transfer of a stable inducing signal. The exogenous addition of 5 μl/ml H_2_O_2_ solution (3% w/v) to these samples resulted in increased fluorescence accumulation (from ≈2.37 of control to 3.25 +/- 0.2 normalized ROS production), arguing against the lower value arising from complete reaction of the available dye or reduced dye incorporation.

## Discussion

Opportunistic parasitic plants provided the first clear evidence of a redox-based, dynamic chemical network functioning along the plant root surface [23,47,48]. Here we show that this process may be far more widespread across diverse rhizospheres. This redox circuit alters the overall RSA of plants in close proximity to one another, a phenomenon not unlike the density-dependent phenotypic switching observed in many bacteria and some fungi known as quorum sensing [49–51]. However, unlike traditional quorum sensing signals (autoinducers) that are synthesized by individuals, ROS/quinone networks arise from a dynamic chemical reaction that depends on two distinct partners. The growing plant root meristem, which has relatively low cell wall phenol densities [24,35], provides reactive oxygen species, while another more mature root provides the requisite phenols, allowing this process to serve as an active detection system for the presence of neighboring mature root surfaces.

In both *S*. *asiatica* and *A*. *thaliana*, BQ perception leads to an increase in cytoplasmic calcium concentrations in approximately the same time frame (≥ 15 min). Blocking the Ca^2+^ channels with La^3+^ ions attenuates the plant’s sensitivity to BQ exposure. Similarly, the antioxidant quinone TFBQ, which does not generate H_2_O_2_ via the mechanisms described in Fig 1, disrupts both Ca^2+^ dynamics and BQ signaling. While both plants respond to the same chemical signal with an increase of [Ca^2+^]_cyt_, the other biochemical and phenotypic responses are remarkably different between parasites and non-parasites. While in parasites the pathway regulates the vegetative/parasitic transition, in non-parasites the pathway functions in a traditional quorum sensing mode with signal (BQ) perception stimulating ROS production to further enhance signal production. Similar autocatalytic behaviors are well-documented components of most QS systems ensuring population-wide responses [50,51].

**Fig 7:**
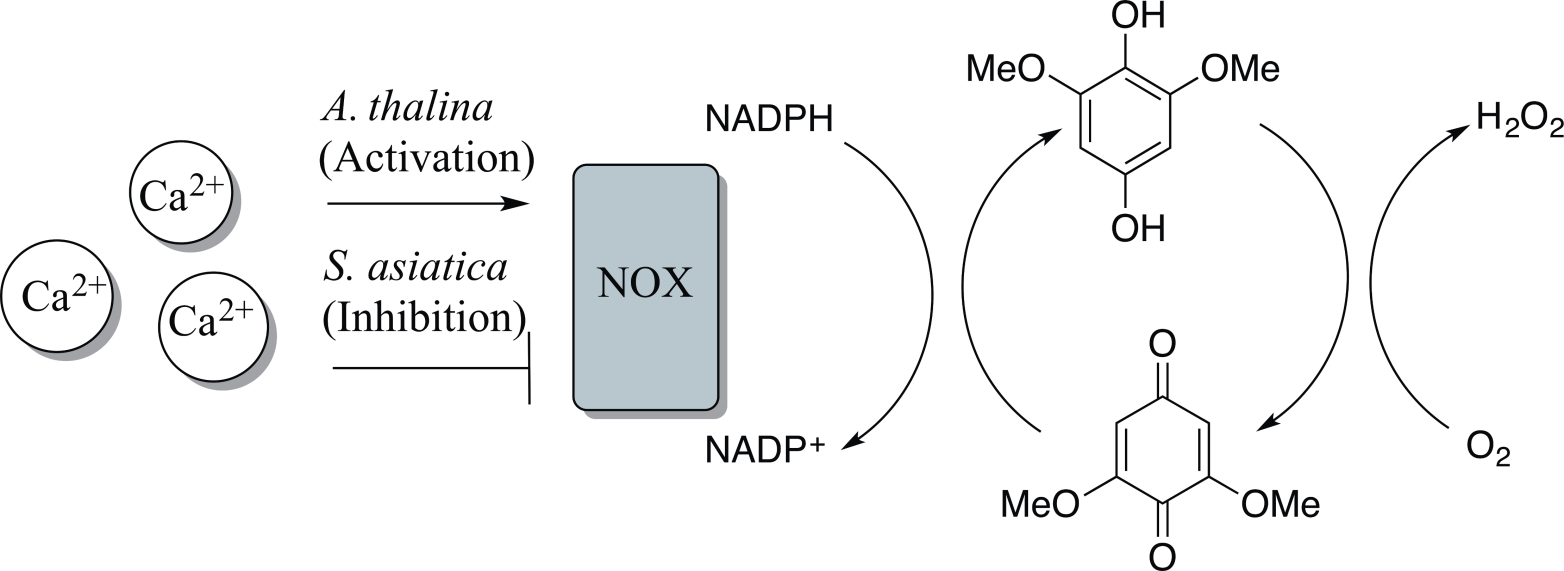
Proposed role of Ca^2+^ balance in BQ regulated root recognition in parasitic and non-parasitic plants. DMBQ reduction by plant’s NADPH- oxidase is needed to maintain the production of hydrogen peroxide via redox cycling. Ca^2+^ assisted NADPH down-regulation of NADPH oxidase in *Sriga* leads to decrease the rate of hydrogen production while up-regulation of the enzyme in *A*. *thaliana* leads to rise in ROS concentration along the root surface that ultimately is perceived as a signal to shut down the root growth.

Detailed examination of the reaction mechanism of ROS generation in the presence of DMBQ can explain these differences (Fig 7). NADH readily reduces quinones to hydroquinones via hydride transfer reactions [52]. Hydroquinones are oxidized by molecular oxygen in a reaction pathway that involves semiquinone and superoxide radical intermediates and results in the production of hydrogen peroxide [53–55]. This reaction scheme is indirectly supported by the insensitivity of *A*. *thaliana* to the antioxidant t-butyl benzoquinone (TBBQ) that cannot chemically produce H_2_O_2_ via autoxidation. A BQ’s specificity as a haustorial inducer also correlates with the quinone’s redox potential, with a narrowly defined window between -280 and +20mV [47], consistent with the redox cycling model and providing indirect evidence for an operating quinone-mediated reaction diffusion network.

Up-regulation of NOX enzyme activity, triggered by increases in Ca^2+^ concentration in *Arabidopsis* [38], would increase the rate of hydroquinone production, and subsequently, produce higher H_2_O_2_ concentrations. In this positive feedback loop, even low initial concentrations of the benzoquinone may result in high final H_2_O_2_ concentrations. In soil, metal ions and biotic enzymes, including cell wall catalases, may rapidly decompose H_2_O_2_. However, if hydroquinone regeneration by NADH is maintained, a steady state concentration of H_2_O_2_ around the root surface could persist for extended durations. In *S*. *asiatica*, however, the initial increase in ROS concentration is followed by a slower decay. The hour-long timing under these conditions is consistent with BQ exposure necessary to induce the vegetative/parasite transition in *S*. *asiatica* [19,23,47].

Our recent investigations of the differential expression of *S*. *asiatica* genes following exposure to DMBQ have shown a dramatic decrease in expression of a specific Ca^2+^-regulated NADPH oxidase (*SaNOX1*) during haustorial development [35]. Consistent with the mechanism proposed in Fig 7, reduction of SaNOX1 activity will decrease the rate of DMBQ cycling and ROS generation. While *SaNOX1* expression is down regulated after sufficient BQ exposure, ROS production declines in the parasite faster than the loss of gene expression. How the production of ROS by SaNOX1 is limited in the presence of increased calcium remains unclear but certainly represents a fundamental point of divergence between parasitic and non-parasitic plants. This simple change, and the subsequent variations to the dynamic chemical network in the rhizosphere, allows the parasites to utilize this process to gauge host proximity. Calcium sensitivity in plant-related NOX proteins may be regulated by phosphorylation, suggesting a redox-sensitive kinase could be responsible for this differential regulation [56,57].

The source of the observed calcium influx will be critical to understanding this network, but has yet to be determined as LaCl_3_ is a non-specific inhibitor of calcium channels. Future work will attempt to map both the origin of the observed calcium influx as well as the regulatory elements and organization of these processes in both plants. However, the ability of these channel inhibitors to prevent BQ induced regulation of ROS production, either up or down, places calcium regulation as an early determinant in this response network.

Oxygen is another critical component of the circuit shown in Fig 7. Recent visualizations of oxygen concentration with planar optodes show sharp concentration gradients along the mature plant roots [58]. Accordingly, the plant root may be able to direct the spatial and temporal distribution of both oxidant and reductant, creating an “external metabolism” of functional significance [59] for plant-plant and plant-microbe signaling. Such redox-mediated reaction-diffusion networks can be complex, generating chemical waves, steady state pattern formation, bistability, and oscillations [60,61], and the details of how such processes operate in the rhizosphere can now be further investigated.

This proposed external redox metabolism may be just one of many existing in the rhizosphere. For example, the monocot hosts of *S*. *asiatica* are known to exude specific electron-rich hydroquinones that accumulate at high concentrations along their root surface [48]. The sorghum-derived germination stimulant spatially restricts *S*. *asiatica* seed germination to the host root surface. Given that this electron rich hydroquinone is readily autoxidized, these reactions might be an even more critical part of dynamic reaction-diffusion rhizosphere networks [9].

The autoinducers associated with quorum sensing regulate a wide variety of bacterial phenotypes, including bioluminescence, virulence factor production, biofilm formation, and motility [4], providing physiological benefits to other members of the rhizosphere ranging from pathogenesis to nitrogen fixation in legume-rhizobia mutualisms [62–64]. Many of these behaviors are also regulated by plant phenols, potentially coupling the redox pathways defined here to bacterial behaviors. Further, since both pathogenic and mutualistic *Rhizobium spp* depend on the phenols, the redox circuit may well impact many of these symbiotic behaviors. Much as cell wall fragments [65] and membrane fatty acids [66] serve as secondary signals for coordinating a variety of eukaryotic cellular responses [67], this external redox signaling network may coordinate significant regional architectures broadly across the rhizosphere.

Finally, it should be noted that the chemistry of hydroquinone auto-oxidation has long been exploited in black and white photography to generate high resolution 2D images. These 2D images are generated when silver salt grains, partially activated by exposure to sunlight, are reduced to silver in the presence of hydroquinones. An initially weak signal is amplified several orders of magnitude by two autocatalytic processes: surface-catalyzed silver reduction and hydroquinone autoxidation. At the same time, the spatial propagation of the signal remains tightly regulated due to the high reaction order and presence of inhibitors, such as sulfite, in developing solutions (69). Building on similar chemistry, the 3D spatial resolution around the plant root could be remarkably precise, providing a dynamic chemical ‘movie’ of the complex multi-component rhizosphere dance[68]. Defining reaction networks that regulate the plant’s second genome are even more necessary and invaluable in this time of our rapidly changing physical climate.
